# Supplementary material for: Patterns of Intron Gain and Loss in Fungi
Source: PLoS Biol. 2004 Nov 30;2(12):e422. doi: 10.1371/journal.pbio.0020422 (PMC532390; doi:10.1371/journal.pbio.0020422)
Supplement: Table S1 — Also available at http://genes.mit.edu/NielsenEtAl/. (4.3 MB ZIP). [file pbio.0020422.st001.zip › NielsenEtAl/html/314.html]

AN6266.1.NCU01503.1.MG01087.1.FG04403.1


```
 CLUSTAL W (1.82) Multiple Sequence Alignments - Introns Inserted


Sequence 1: NCU01503.1	817 aa
Sequence 2: MG01087.1	810 aa
Sequence 3: FG04403.1	815 aa
Sequence 4: AN6266.1	810 aa
Alignment Length: 842 aa
Number Identitical Residues: 374 aa
Alignment Score (without introns) 19204


MG01087.1 	-MPSAVAG--HRPTTKVGNKPFKSRKATKGAIRTLAKG1KVP-TEKGQRKTQHQQVMSKL
NCU01503.1	-MPGAVSGHSHRPTTKASNKPFKSRHSTKGALRNAAKG1KVA-EERGVRKTPHQQVMSKF
FG04403.1 	-MPTAVAHS-HRPTTKVTHKSFKSRAASKHELRDRAKG1RVS-NEKGNRKTPHQQVMSKF
AN6266.1  	MAPTSTVQHHHRSTTKTTHKPYKSGHASKRALKDQAKG1KVERAERGTRKTPHQQLMSKL
          	  * :.    **.***. :*.:**  ::*  ::  *** :*   *:* *** ***:***:

MG01087.1 	DRRNQSRQKLLNKQREHRSDNQVFAGRDGAPRNVAVIPLCADGDAADAIRSLNLSLDIET
NCU01503.1	DRRNRNKQLQLQKHKEHLQETSIFAGKDGAPRNVAVIPLCSDGDATEAIKSLNASIDIET
FG04403.1 	DRRNQAKQARLNKHKEQVKDTLVFAGKDGAPRHVALIPLCAGTDLNATIKQLNSSVDIED
AN6266.1  	DRRNQARQKQQNKQQERAQAISIFSGQNGAPRHVAIVPLSIDIDTTAIIKALNESVDVTS
          	****: :*   :*::*: .   :*:*::****:**::**. . *    *: ** *:*:  

MG01087.1 	EVSPGCT-RVSVDRFKQKLAYFPVVRDLATCLDAARVADFVVLVLSAEVEVDQLGELILR
NCU01503.1	EVPEGAF-RVPVDRFKQKLQYFPLKRDLTACLDAARVADYVVLVLSAQEEVDELGELILR
FG04403.1 	NADGGNY-RVTVDRFKQKLQYIPIDRDLNACLDAARVADFVIVVLSASVEVDGLGELILR
AN6266.1  	DVFPDTISRVRIDRFRQNLQYIPAKYDLMSALDACRMADFVVLAISAHLEVEEQGELLLR
          	:.  .  :** :***:*:* *:*   ** :.***.*:**:*::.:**  **:  ***:**

MG01087.1 	SVESQGVSTVFTVVQGLNKVEPVKQRLSIVDSLKSYIKYYHPDQDKIYSLDNRQECSNLM
NCU01503.1	SVESQGLSTLFTVVQGLNKIEPQKQRLSVLSSLKSYITHFHPEQDKVFSLDNRQECSNLM
FG04403.1 	SVESQGMSTLFTVVEGLDKVEPAKQRQSVLGSLRSFITHFHPEQEKLYSIENRQECANLM
AN6266.1  	SIEGQGVSNVIAVVQGLDKINPPKKRPQVAGSLKSFLNHFYPSIDKVLSIDSRQECSNAI
          	*:*.**:*.:::**:**:*::* *:* .: .**:*::.:::*. :*: *::.****:* :

MG01087.1 	RSVCSTTPKGVVWRDERSWMLVEDVQFPASES---DLTVVTGVVRGKGLKADRLVQVGDW
NCU01503.1	RSLCSTTPKGIRWRDERSWMLVEEVKFGES-------TVLTGVVRGKGLKADRLVQVGDW
FG04403.1 	RSLCNTTPKGIRWRDDRSWMLAEDIKFAYSES---EPTVITGIVRGKGLKADRLVQVGDW
AN6266.1  	RSLCTATPKGIRWRDDRSWMLIEDVKWPESASPEVDDVVLTGVVRGKGLKADRLVHVPGW
          	**:*.:****: ***:***** *::::  * :.. . .*:**:************:* .*

MG01087.1 	GTFQIEKIMAAPLSTKKAKEGS-MAVDDAPSQEPLDVPTDDRDDLDELAPEEIVMEDD--
NCU01503.1	GTFQVEKITAAPLARRKRGEEG-MNVEEG-AEEILDQPTDDRDDLVDLAPEEVRMEDDDD
FG04403.1 	GTFQIEKIESAPLPKQVKKKGEEMTIDPEEAGKVLDEPSADRDELVELAPEEVMMDAD--
AN6266.1  	GDFQIDSITAAPLPTARSKRDDAMNVDESEAPQLLDSPSADRDDLATVAPEEIEMEDD--
          	* **::.* :***.     .   * ::   : : ** *: ***:*  :****: *: *  

MG01087.1 	DEMDDAQTSASAPTKKGVLLDDHHYFSDDEEGQPQIRKRLPKGTSKYQSAWYLDDGVSDS
NCU01503.1	DEMNDGMTTMTT-SKKGVLLDDHHYFSEEEPEAYTAPKKLPKGTSKYQAAWYLDDDVSDS
FG04403.1 	----VAEMEADASEKKGVLLDDHHYFTDEEAEAAKIKKRVPKGTSNYQSAWYLED-VSDS
AN6266.1  	------IESMPDTDRKGVLLDDHHYFSDDDDHIPAKPKRLPKGTSEYQSHWFLDD-VSDS
          	            . :***********::::       *::*****:**: *:*:* ****

MG01087.1 	GSDLEDLDMEDDAQSEAAPRPEDGVEGMAMDAQTEYGASEYPQSE-MFVEPNEDEDAKQL
NCU01503.1	GSDMEEDDEMEDDDEEEAARPEDGEEGLAGYAPTEAGQTEYPQTEGGFIQPDADEDAKQL
FG04403.1 	ESDMEDYEMPDDD--AEQARPEDGIEGYAPAPGTEGAPSEYPQSE-MMIEPDEEEDLKQL
AN6266.1  	GSDVMDDDDEEDMQMDVSGAPEDGVFPDNHDAMTEGGPSEYPQSE-MFLDPSPEDEAQQL
          	 **: : :  :* .      ****       . ** . :****:*  :::*. ::: :**

MG01087.1 	EAYRASRRDEAEDDKEFPDEIELHPNVLARERLARYRGLKSLRTSKWEEEEDRAHEPEDW
NCU01503.1	EAFRKQKRDEAQDDLEFPDEIELHPHVLARERLAKYRGLKSLRSSPWQEDEDRAYQPEDW
FG04403.1 	EQYRAGKRTEEEDDMEFPDEIELHPGVLARERLARYRGLKSLRTSRWEESEDRAHEPEDW
AN6266.1  	EEYRASRRNEAKDDLEFPDEIELHPNVLARERLARYRGMKSLKTSPWNTAEDRPHEPEDW
          	* :*  :* * :** ********** ********:***:***::* *:  ***.::****

MG01087.1 	RRLLQVPDYNGSKSRCTREALVGGVAPGTRVAVHLKGVPASVAASYDGRKPITMFSLLRH
NCU01503.1	ERLLKIQDYQASKIRASREALVGGVAPGTRVHVYLKDVPATIQQSYNPSQPVTLFSLLRH
FG04403.1 	RRLLQVPNYQSSKSQATREALVGGVEPGTRVHIYIKGISPTQEKTYDPKKPLTLFSLLRH
AN6266.1  	RRLLQFGDYKGSKNRILREALVGGVNPGTRVDVHLRAVPALLR---NRPQPLSLFSLLRH
          	.***:. :*:.** :  ******** ***** :::: :..      :  :*:::******

MG01087.1 	ENKQSVVNFLINLSSEFPRSLKAKEELVIQYGPRRLVINPLFSQGGNTPNDVHKYGRFLH
NCU01503.1	EYKKTAVNFLINLSADAAAPIKAKEELIIQCGPRRFVINPLFSQGGNTPNDVHKYCRYLH
FG04403.1 	ENKKTAVNYLFNLSSDFTQSIKAKEELIVQCGPRRMVIKPLYSQPGQTPNDVHKYCRFIH
AN6266.1  	EHKHTVVNINMHLNAAVEEPIKSKQELIVQYGPRRLIVNPVFSSADNTPNNVHKFDRFLH
          	* *::.**  ::*.:    .:*:*:**::* ****::::*::*. .:***:***: *::*

MG01087.1 	PGQSAVATFVGPVIWGPVPALFFKKQ--------------------EGSDDLSLTLVGTG
NCU01503.1	PGQSAVATFTGPVTWGAVPTLFFKRQVP----------GSVLDEDGEPVSNLPLSLVATG
FG04403.1 	PGQSAIATFMGPLTWGAVPVLFFKRTTA----------EDVEKNEEEDGPHIGMSLIGTG
AN6266.1  	PGRSAVATWIGPLTWGSVPVLVFKSRKTSADPEILDSADAPESTTAVGFSTSTLELIGTG
          	**:**:**: **: **.**.*.**   .::...  .::    .          : *:.**

MG01087.1 	TALPPSTGRVVAKRVILTGHPYHIHKKIVTIRYMFFNREDVEWFKALPLWTRRGRSGYFK
NCU01503.1	TALPPSTSRVVAKRAILTGHPYHIHKKIVTIRYMFFNREDVEWFKALPLWTRRGRSGFIK
FG04403.1 	TAIPPSTSRVIAKRIILTGHPYHIHKRIVTIRYMFFNREDVEWFKALPLWTKRGRSGFMK
AN6266.1  	TVVAPDPTRVVAKRAIITGHPYKIHKRVVTVRYMFFNAEDVNWFKALQLWTRRGRSGFIK
          	*.:.*.. **:*** *:*****:***::**:****** ***:***** ***:*****::*

MG01087.1 	EALGTHGYFKATFDGRINPQDSVGVSLYKRVWPRKSVPFRGELVGAEEAPKLVEAGAMDE
NCU01503.1	EALGTHGYFKATFDGRINPQDSVGVSLYKRVWPRSAKQLGAPLLDAAALQQEVDEDAMDA
FG04403.1 	EPLGTHGYFKATFDGRINPQDSIGVSLYKRVWPRTAVPVEGPLLDIATSRIQADGDMMMD
AN6266.1  	ESLGTHGYFKCTFDGKINPQDSVGISLYKRVFPRKAR----ALEEVAN------------
          	*.********.****:******:*:******:**.:      *                 

MG01087.1 	DIA
NCU01503.1	E--
FG04403.1 	DQA
AN6266.1  	---
          	
```
